# Socioeconomic conditions exacerbated by the coronavirus in the Eastern Cape province of South Africa

**DOI:** 10.3389/fpubh.2025.1526309

**Published:** 2025-04-22

**Authors:** Tlou D. Raphela, Sinoyolo Tjantjies

**Affiliations:** Disaster Management Training and Education Centre for Africa, University of the Free State, Bloemfontein, South Africa

**Keywords:** coronavirus, socioeconomic, vulnerabilities, pandemics, viruses

## Abstract

**Introduction:**

South Africa, like most developing countries, made some efforts to attain the Sustainable Development Goals by endeavoring to reduce individuals' susceptibility to socio-economic hardships. However, the COVID-19 pandemic eroded most of those efforts. In this seemingly helpless situation, it is apparent that the coronavirus has compromised the commendable strides made toward achieving some of the efforts toward attaining some of these Sustainable Development Goals. This article, therefore, analyses the socioeconomic conditions exacerbated by COVID-19 on the East Coast of South Africa.

**Methodology:**

The study adopted a quantitative research-method approach to quantify this socio-economic disparities and applied, descriptive statistics, chi-squared test of independence, and regression to analyse the data collected using a semi-structured questionnaire survey at a rural community in the Eastern Cape of South Africa. Simple random sampling was adopted for this study and Statistical Package for Social Scientists was used to analyse the data collected.

**Results:**

There was no significant relationship when the question of whether the municipality has adequately implemented measures to ensure the socio-economic protection of communities was regressed with other 3 Likert scale questions (χ^2^ = 95.98; df = 77; *P* = 0.07; *R*^2^ = 38.0%). A series of Chi-square tests did not reveal any statistically significant difference in five demographic variables and the variables they were correlated with (*P* > 0.05). However, there was a statistically significant difference between most questions relating to the effectiveness of the COVID-19 response strategies the municipality employed and participants' response to unmet community social needs (X^2^ = 35.754; df = 14; *P* = 0.001).

**Discussions and conclusions:**

This study, therefore, revealed that social significance does not necessarily mean statistically significant following the results of the insignificant chi-squared test for the socio-economic and demographic variables. This study also sheds light on the profound impact of the COVID-19 pandemic on the socioeconomic landscape of the East Coast of South Africa. Despite commendable strides toward achieving Sustainable Development Goals aimed at reducing vulnerabilities to socio-economic hardships, the pandemic has presented daunting challenges, disrupting progress and exacerbating existing inequalities coupled with efforts of the study Municipalities strategies.

## 1 Introduction

The COVID-19 pandemic has had significant socioeconomic impacts globally, and South Africa, like many other countries, has experienced these effects acutely (1, 2). Pandemic has exacerbated poverty levels, increased unemployment rates, disrupted livelihoods, and widened existing inequalities in many regions, including the Eastern Cape Province (3). Sustainable Development Goal (SDG) 1 aims to end poverty in all its forms everywhere, and addressing the socioeconomic challenges exacerbated by the pandemic aligns directly with this goal. SDG 1 emphasizes the need for targeted interventions to support those most affected by poverty, including through social protection measures, employment generation, and sustainable economic development strategies. Buheji et al. (2) reported that global governments enforced stringent lockdowns that impacted the socioeconomic conditions of communities' globally to curb the spread of the COVID-19 pandemic. Such restrictive lockdown measures negate livelihood requirements (4). Due to no fault of their own, poorer communities were thus unable to adhere to social isolation measures (5). Indeed, Buheji et al. (2) reported that COVID-19 could retrograde the global poverty levels to those documented 30 years ago. Thus, the turmoil effect of COVID-19 threatens the United Nations Sustainable Development Goal (SDGs) for poverty alleviating arguments. Pillai et al. (6) supported the above arguments, stating that COVID-19 has debilitated even the strongest eco nomic regions. The cascading impact of COVID-19 included reduced human capital, permanent closure of small to medium enterprises, rise in unemployment, amongst other socioeconomic variables (7). Given the ever-changing risk environment of COVID-19 and its ripple effect on all spheres of socioeconomic activities, it cannot be overlooked that an individual's livelihood and access to capital are influenced by shocks (8, 9). An economic recession triggered by COVID-19 exposes how biological hazards such as viruses have the potential to manifest into a socioeconomic disaster. Summatively, COVID-19 plays a catalytic role in worsening vulnerabilities amongst people, economies, and systems. Inevitably, the most affected are undoubtedly the vulnerable population who are likely to experience difficulties recovering from the socioeconomic impacts of COVID-19 (10). Khim (9) points out that some socioeconomic impacts of COVID-19 also include the worsening of prevailing food insecurity amongst vulnerable communities. Social isolation measures have ensued, reducing social mobility through a scaled-down workforce and school closure. Amidst the pandemic, school closures endeavored to prevent infection propagation. The disruption in the education system has inadvertently increased childcare costs amongst families caring for young children. Baldwin and di Mauro (11) acknowledges that although COVID-19 has evoked socioeconomic malaise in many global communities, it cannot be unheeded that South Africa is already rife with social ills and inequality. Moreover, COVID-19 exposed the fault lines in many government disciplines, more specifically, Disaster Relief Management (11). South Africa is a country ravaged by poverty, unemployment, and inequalities (12). These inequalities are apparent in the service delivery of housing, sanitation, education, and health care. Given the above, this study aims to shed light on the socioeconomic conditions exacerbated by COVID-19 in the Walmer Township in the Nelson Mandela Bay Municipality located in Port Elizabeth in the Eastern Cape Province of South Africa. This study aim will be realized by quantifying the socio-economic disparities brought about by the COVID-19 pandemic to supply relevant stakeholders with quantified actionable insight and to give quantifiable recommendations to the policy makers. Indeed, a study by Morgan (62) reported that decision-makers and policymakers can use quantitative approaches to guide outbreaks responses before COVID-19. Another recent study reported that to get herd immunity during COVID-19 policy makers needed total measurable data (63). This study will answer the following questions:

- To what extent did the Nelson Mandela Bay Municipality prepare for biological hazards such as the coronavirus?
- What social conditions are exacerbated by COVID-19 in Walmer Township?
- What economic conditions are exacerbated by COVID-19 in Walmer Township
- What are the perceptions surrounding the adequacy of socioeconomic protection of the Walmer community by the government amidst the COVID-19 pandemic.

## 2 Materials and methods

### 2.1 Background of the study area

**Figure 1:**
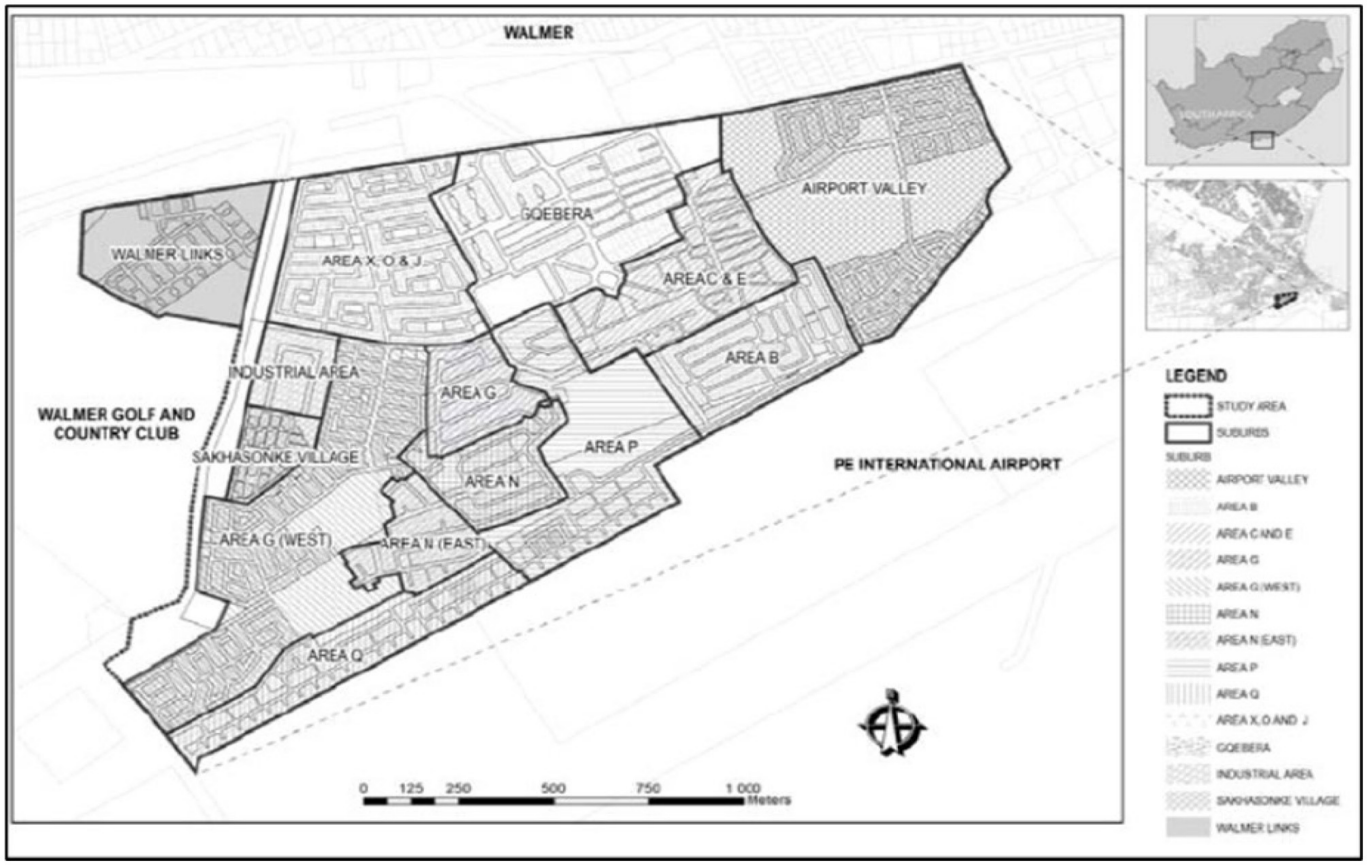
The location of the study area Walmer relative to the Port Elizabeth Airport in the Nelson Mandela Bay Municipality **(top)**, the bottom map shows Ward 4, where the study area is situated. Source: SRK Consulting (2015).

The study placed special focus on the Walmer Township within the Nelson Mandela Bay Municipality in Port Elizabeth in the Eastern Cape Province of South Africa (Figure 1). South Africa's liberation struggle, the most protracted in the subcontinent, was distinguished by the extent to which socio-economic issues played out in the informal settlements, and especially in the rural provinces (13). According to Njigana (14), the Walmer Township, also known as Gqebera, sprung up during the apartheid years. Since then, South Africa's democratic government has immersed itself in dismantling the infrastructure backlog in marginalized communities. However, the Walmer township predominately consists of informal settlements with a very high population density. The Walmer Township is the poorest residential area in Port Elizabeth, with an approximate population of more than 65,000 people (15), with an average of 10 people residing in a household (14). Residents of the Walmer Township are plagued by socioeconomic issues such as high unemployment, malnutrition, crime and health ailments such as HIV and AIDS. In light of the COVID-19 scourge, social and physical distancing is used to prevent virus carriage to the vulnerable population. Having discussed the Walmer Township's housing informality, note that densely populated areas, such as the Walmer Township, cannot manage health hazards (16). Considering that the harsh lockdown brought economic activity to a near-standstill. It is worth noting that even before COVID-19, the Walmer residents were grappling with financial fragility and were still heavily reliant on informal economic activities. Along with the rising death rate, so are the unemployment rates rising. Most of all, efforts to contain the virus's spread place many low socioeconomic communities in a serious dilemma, whereby residents must choose whether to risk getting infected or going hungry (16). Worst of all, overpopulation in the community has reduced food production. The issue of high unemployment amongst the people of working age in the Walmer Township cannot be overlooked.

The high influx of people migrating into the Walmer Township has limited access to basic services such as electricity, water, and sanitation. Currently, many Walmer residents walk long distances to collect water and use the bucket system; thus, water supply and sanitation remain unmet basic services. In the times of the COVID-19 pandemic, where the governments are encouraging proper hygiene and washing of hands regularly, this is a serious problem. In keeping with previous arguments, Chirisa et al. (16) unpack the dire consequences of squalid living conditions, particularly inaccessibility to clean water and decent sanitation facilities. Hishan et al. (17) recognize that the community's susceptibility to COVID-19 is aggravated by its inaccessibility to readily available clean water and poor hygiene. Zoning into the Walmer Township, Chirisa et al. (16) argue that the community's use of communal sanitation facilities and taps increases their vulnerability to infection, in particular the COVID-19 infection. In addition, Walmer Township is situated near industrial sites (sources of pollution), namely Wells Estate and Port Elizabeth International Airport. The community is susceptible to socio-environmental hazards, and because of the airport, the vulnerability of this community to COVID-19 is increased as the is distributed by people moving from one place to another, more especially across borders. Above all, the Walmer residents are burdened by ill health, namely respiratory infections that include respiratory tract infections such as rapid breathing, chest pains, fever, bronchitis, tuberculosis, and or pneumonia (18). In light of the above, Teare et al. (18) deduce that since poor living standards are determinants of ill health, treating an individual's ailments must be coupled with interventions that address the conditions that caused the individual's ill health. Moreover, residents of the Walmer Township are riddled with low income, poverty, low-quality housing, overcrowding, ill health, and high levels of debt (18). As stated by the WHO (19), healthy housing promotes good health and mental health wellbeing.

### 2.2 Methodology

To address the research problem and objectives, this study used a quantitative research approach. Socioeconomic inequalities are inherently complex, involving both measurable outcomes and subjective experiences. The quantitative component, collected through the semi-structured questionnaires, provides statistical insights into patterns and disparities. However, these numerical findings are insufficient to capture the lived realities and contextual nuances of the affected populations, this is supplemented by the open-ended questions on the questionnaire which were also analyzed quantitatively.

This study employed a quantitative research approach and adopted a post-positivist philosophical worldview to cater to the one open-ended question on the questionnaire. The study collected data without manipulating any research variables and was random in nature. The study collected data using a questionnaire with close-ended questions which were analyzed quantitatively and one open-ended question which was analyzed thematically. Given that COVID-19 is a new phenomenon that has ensnared global communities, the quantitative research design allowed the research to corroborate findings generated within the study and to be able to compare quantified measures with other pandemic studies. The study targeted the Walmer Township and obtained the population size of 70,000 for this Township from the Statistics South Africa offices in East London, Eastern Cape Province of South Africa (20). The participants were chosen through a random sampling selection process from this population size of 70,000. Since this study is quantitative in nature sample size was also determined with a confidence level of 95% and a margin of error of 5% using a Cochran formula for finite populations as follows:


$$\begin{array}{l} n=\frac{Z^{2}\;*P\;*\;\left(1-P\right)}{e^{2}}\; \end{array}$$

Where

*n* is the required sample size for an infinite population = 70,000

Z is the Z-score corresponding to the desired confidence level (chosen confidence level for the study is 95%)

*P* is the estimated proportion of the population with the characteristic of interest (for this study is 0.5, as it provides the maximum variability).

*e* is the margin of error (0.05 for a 5% margin of error).

As the study obtained a finite population size for the study community, we adjusted *n* using *n*_*adjusted*_ *where* *n*_*adjusted*_ *is* the final sample size for the finite population as follows:


$$\begin{array}{l} n_{adjusted}=\frac{n}{1+\;\frac{n-\;1}{N}} \end{array}$$

The sample size for the study community was calculated to be 384. Despite the calculated sample size, the researcher has opted to reduce the sample size to 100 participants for this study. Justifying the above choice, literature reported that 100 participants are a large enough sample to gather statistically significant data (21). Along with achieving sound statistical results, a sample size of 100 will allow a comprehensive understanding of the new phenomenon. Summatively, an unreasonably large sample size exposes participants to avoidable risks and wastes resources and the time of participants and investigators. The study used semi-structured questionnaires as a primary data-collection tool. Leedy and Ormrod (22) deeply explores questionnaires stating that questionnaires are a set of standardized questions used to gather data about ideas, attitudes, feelings, and perceptions. This study consisted of demographic questions that gathered participants' demographic data such as age, marital status, and educational background. Also, the questionnaire consisted of a combination of closed-ended and open-ended questions. Before the questionnaires were administration, participants had been physically contacted by the researcher, and information about the study was conveyed. Additionally, participants have been allowed to ask questions about the study and make an informed decision by receiving a hard copy of the information leaflet. Before the questionnaire dissemination, each participant was issued an information sheet, which provided a brief overview of the study and explained the purpose of the inquiry. Also, all participants have been issued a written consent form. For this study, the researcher utilized thematic analysis with the aid of Microsoft Excel 2010 Version to analyse the one open-ended question for this study.

Through thematic analysis, the researcher unpacked emergent themes. The study analyzed closed-ended questions in the questionnaire using descriptive and inferential statistics to interpret and analyse the quantitative data. Given that descriptive statistics in the quantitative paradigm summarize and describe the dataset, the researcher has prepared tables and visualized the data using IBM Statistical Package for Social Scientists (SPSS version 29). Microsoft Office Excel program was used for capturing, cleaning and coding the data before importing it into the SPSS for statistical analysis. The study was guided by principles of beneficence, anonymity, and non-maleficence. Ethical approval for this study was obtained from the General Human Ethics Committee (GHREC) of the University of the Free State, with an ethical clearance number of UFS-HSD2021/1150/21. Moreover, the participants were not coerced to partake in the study. All participants exercised their power of choice. After the research participants had assimilated essential information about the study, the study obtained informed consent from all participants.

#### 2.2.1 Statistical analysis

In this study, all statistical tests were run using SPSS, and the *P*-value was set at *P* ≤ 0.05. The study gauged the preparedness of the NMBM for COVID-19, by asking four Likert scale questions separately and analyzing them as one using an ordinal regression because the data were not normally distributed even after log transformation. The researcher set responses to the question of whether the NMBM has adequately implemented measures to ensure the socio-economic protection of communities in the municipal area (as the dependent variable), whereas the responses to the other three questions were set as the covariates of the model. To assess the social conditions exacerbated by COVID-19, the study applied a series of Chi-Squared tests of independence to gauge relationships between variables. The study applied four separate Chi-Squared tests of independence between some critical demographic variables (gender, marital status, age, and source of income) and other key social conditions questions to deduce relations between these variables. In addition, the study also correlated two other important key social condition variables with each other to assess the relationship that might exist. To address the economic conditions exacerbated by COVID-19 in the Walmer community, the researcher applied a series of Chi-square tests of independence between variables to deduce the relationship between these responses. The only open-ended question of the study was analyzed thematically using Microsoft Excel 2010 version.

## 3 Results

### 3.1 Introduction

This study is based on 100 participants instead of 384 participants determined using the Cochran formula (See methodology section above). While the researchers planned to collect the determined sample size as the study is most people chose not to participate in the study as they were allowed to do so through to abide by the University ethical conduct and information sheet that governed the study. This could be because people were possibly COVID-19 fatigued and did not want to answer questions that were related to the COVID-19 pandemic. Indeed, studies around the world have reported communities to be COVID-19 fatigued (23–25). This study acknowledges the limitation of this reduced sample size and how it could have affected the conclusions of this study. However, a sample size of 100 for a quantitative study has been reported to give good statistical inferences (26, 27). Nevertheless, considering this limitation this study recommendation will not be applied to any other community.

### 3.2 Level of preparedness

The ordinal regression results did not show a significant relationship when the question of whether the NMBM has adequately implemented measures to ensure the socio-economic protection of communities in the Municipality (dependent variable) was regressed with other 3 Likert scale questions (χ^2^ = 95.98; df = 77; *P* = 0.07; *R*^2^ = 38.0%). However, significant differences were found for the dependent variable, and neither agree nor agree, slightly agree, slightly disagree only (Table 1). The significant parameters are shown in bold. Therefore, the regression results have shed light on COVID-19 preparedness deficits as far as the Walmer community. Responses such as “neither agree nor disagree,” “slightly agree,” and “slightly disagree” were identified as having notable distinctions in these results, indicating nuanced variations in perception that may require further exploration. These findings highlight the complexity of community perceptions and suggest that practical efforts should focus on understanding the subtleties in stakeholder opinions to refine socio-economic protection measures effectively.

**Table 1:** Output of regression analysis for the Likert scale questions to assess whether the NMBM has adequately implemented measures to ensure the socioeconomic protection of communities in the Municipality.

**Parameters**	**Estimates**	**Std errors**	***P*-values**
Socio-economic protection: neither agree nor agree	**−3.669**	**0.698**	**< 001**
Socio-economic protection: neither agree nor agree	**−2.264**	**0.587**	**< 001**
Socio-economic protection: neither agree nor agree	**−2.137**	**0.581**	**< 001**
Socio-economic protection: neither agree nor agree	−0.917	0.544	0.092
Clean drinking water	−0.086	0.164	0.600
Crime increases	−0.264	0.419	0.529
Substance abuse increases	−0.276	0.272	0.309

Source: Study Data SPSS Software. Statistical values are shown in bold.

### 3.3 Social conditions exacerbated by the COVID-19 pandemic

**Figure 2:**
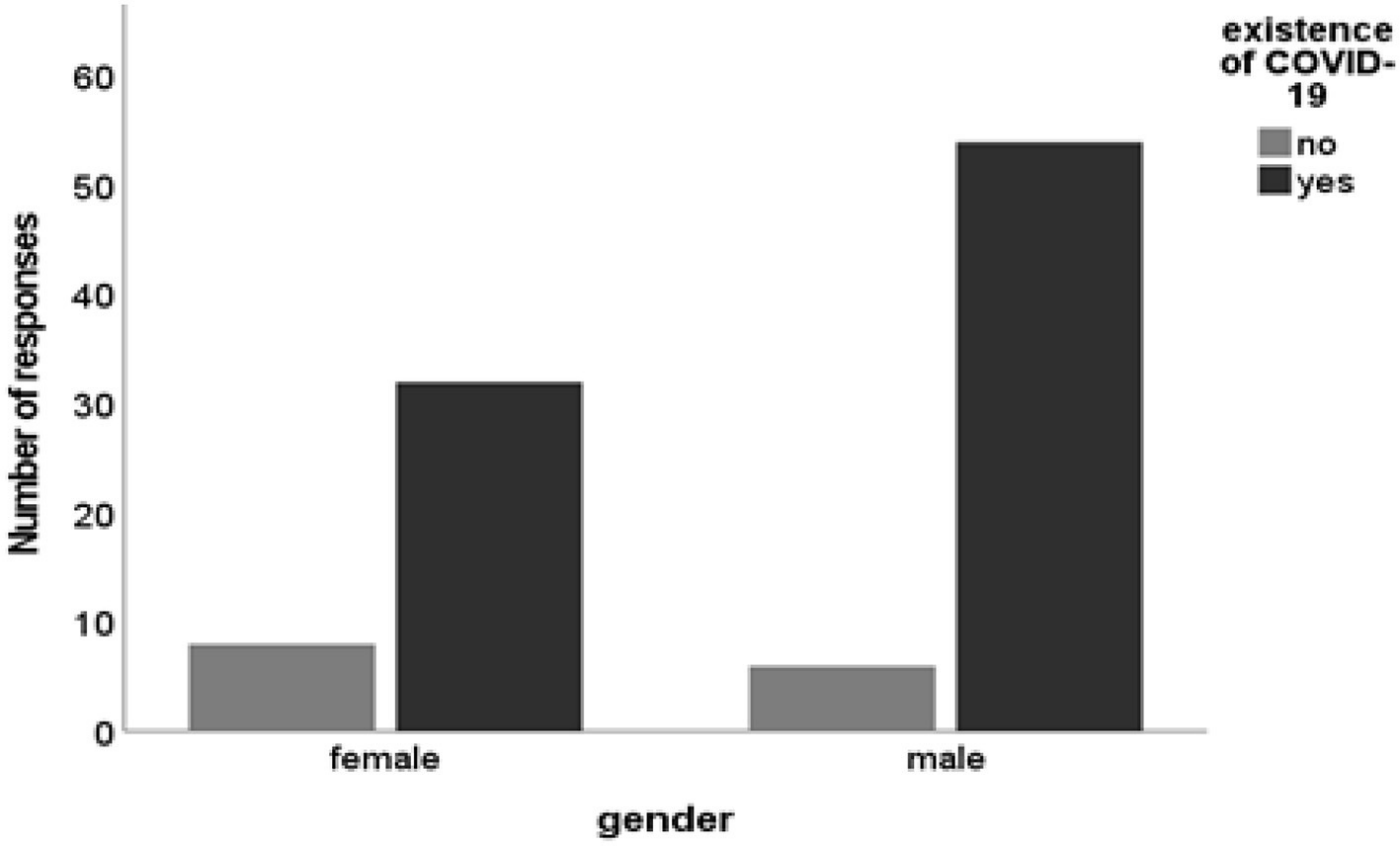
A cross-tabulation between the participants' gender and beliefs on the existence of COVID-19. Source: Study Data SPSS Software.

There was no statistically significant difference between gender and participants' responses when they were asked about the existence of COVID-19 (χ^2^ = 1.993; df = 1; *P* = 0.158. However, the highest number of males reported that they believe COVID-19 exists as compared to females (Figure 2). Additionally, the highest number of males reported that they believe COVID-19 exists as compared to females. Fourteen (12)%, of the participants indicated that they did not believe that COVID-19 exists. These findings suggest potential gender-based trends in perception, even though they were not statistically significant, and highlight a minority group with disbelief that warrants further exploration especially since COVID-19 was novel.

**Figure 3:**
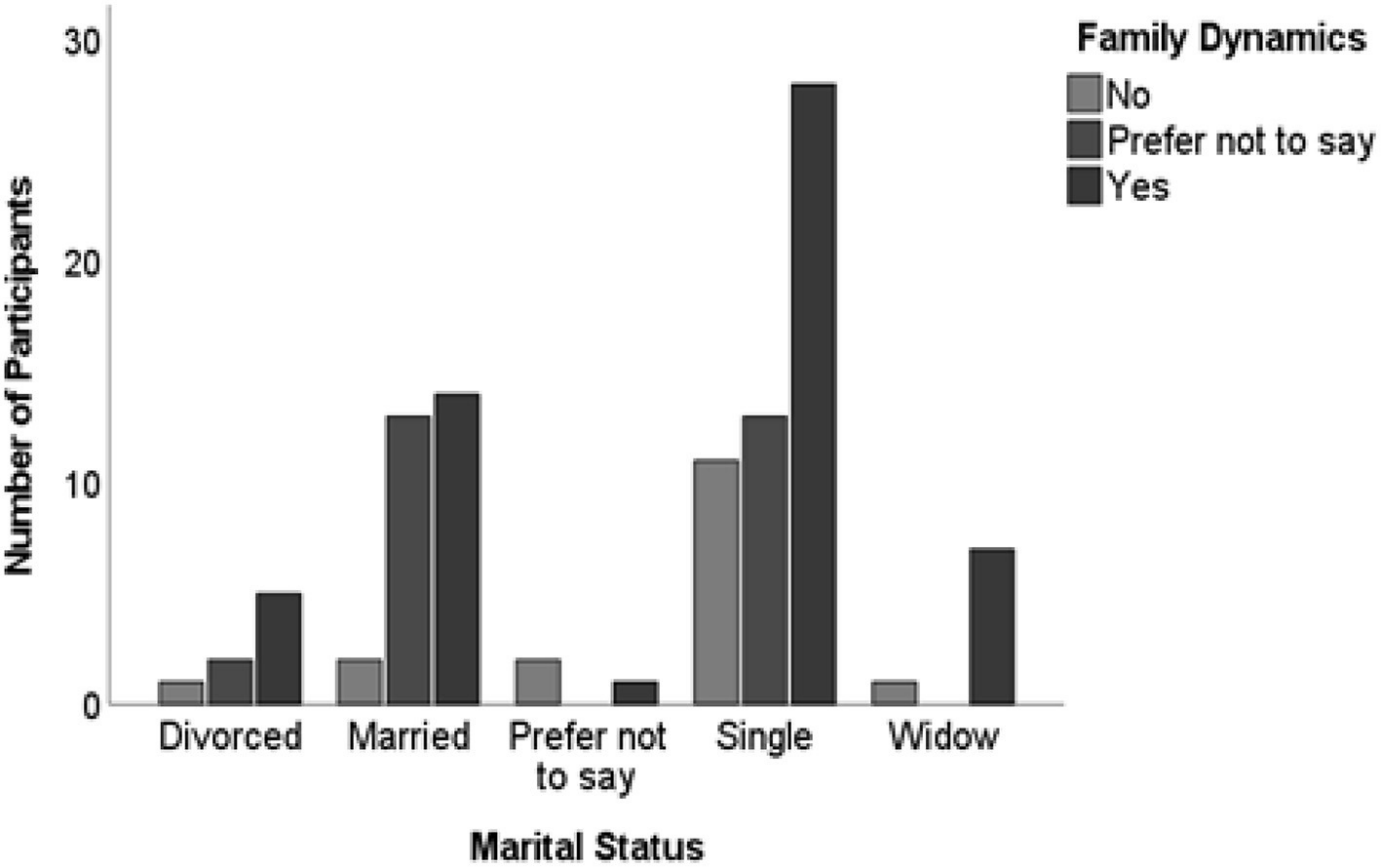
A cross-tabulation between the participants' marital status and participants' response to COVID-19′s impact on family dynamics. Source: Study Data SPSS Software.

The Chi-square test did not reveal any statistically significant difference between marital status and participant's response to COVID-19′s impact on family dynamics and community relations (χ^2^ = 15.144; df = 8; *P* = 0.056. However, most of the participants who mentioned that they were single had reported that COVID-19 had affected family dynamics and community relations (Figure 3). It is practically noteworthy that a considerable proportion of participants identifying as single reported experiencing effects of COVID-19 on these dynamics. This finding, while not statistically conclusive, suggests potential trends that warrant further exploration to better understand the nuanced ways in which marital status might influence perceptions of social and familial impacts during the pandemic especially since studies have reported marital status to be an issue during disasters (28–30). However, the findings that most single people have reported the effect is consistent with studies that reported single people to be more affected during disasters (29, 31, 64).

**Figure 4:**
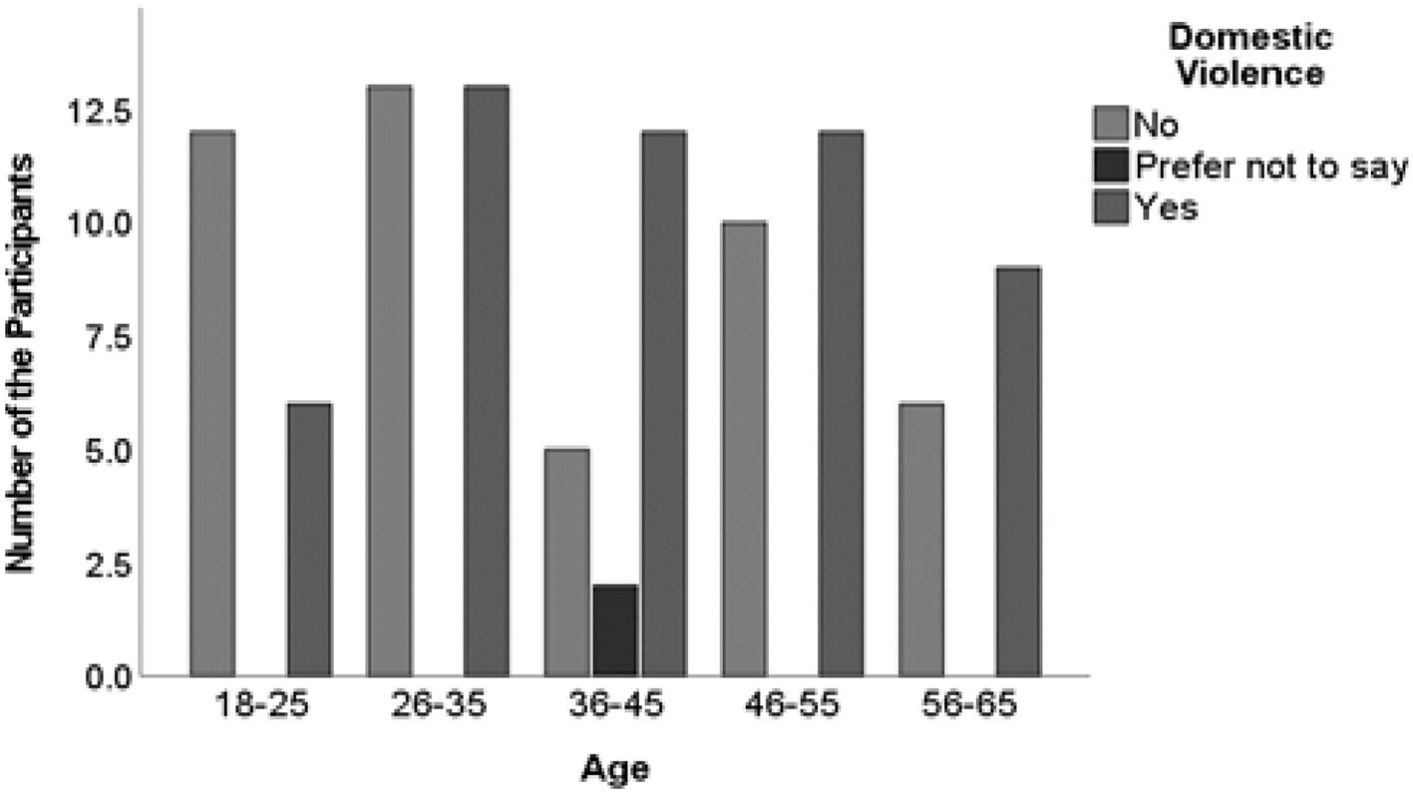
A cross-tabulation between the participants' age group and participants' response to COVID-19′s impact on domestic violence. Source: Study Data SPSS Software.

There was also no statistically significant difference between the age group of participants and participants' response to COVID-19′s impact on domestic violence (χ^2^ = 13.900; df = 8; *P* = 0.084. However, irrespective of the participant's age group, many of the participants reported that COVID-19 had impacted domestic violence (Figure 4). This finding suggests that age group did not play a decisive role in shaping participants' perceptions of the issue. However, regardless of age, many participants reported that COVID-19 had impacted domestic violence, highlighting the broader relevance of this issue across different age demographics. This practical insight emphasizes the need for inclusive interventions and support mechanisms that address domestic violence universally, without age-specific targeting.

There was no statistical significance difference between participants' source of income and participants' responses on the varying sources of income and the question of whether this is affecting the mental health of the participants (χ^2^ = 7.585; df = 5; *P* = 0.181). However, many of the participants who were dependent on casual jobs as a source of income indicated yes when asked if the source of income affected their mental health. From a practical perspective, it is noteworthy that many participants relying on casual jobs reported that their source of income negatively impacted their mental health. This suggests that while the relationship may not meet statistical significance thresholds, the experiences of those in casual employment highlight a potentially meaningful trend that warrants consideration in interventions or policies addressing mental health and income stability.

There was also no statistically significant difference between participants' reports on school performance and participants' responses on unconventional learning experiences (χ^2^ = 0.231; df = 4; *P* = 0.994). However, many of the participants indicated yes when asked if COVID-19 had impacted their school performance. In this study, 61% of the participants mentioned that COVID-19 had a bothersome impact on school performance, whereas 39% of the participants mentioned that COVID-19 had no bearing on their school performance. A notable number of participants acknowledged that COVID-19 had impacted their school performance. This highlights the practical significance of external factors like the pandemic, which might influence academic outcomes independently of unconventional learning experiences. Therefore, addressing pandemic-related challenges may be more impactful than focusing solely on unconventional learning practices to improve school performance. There was a statistically significant difference between the question of effective COVID-19 response strategies by the municipality and participants' response to unmet community social needs (χ^2^ = 35.754; df = 14; *P* = 0.001. However, most participants reported “no” to most questions relating to the effectiveness of the COVID-19 response strategies employed by their municipality (Table 2). The participant who selected the other option mentioned that another urgent community social need that remains unaddressed is the employment of residents from the Walmer Township. While exploring the effectiveness of COVID-19 response strategies, participants were permitted to select multiple responses. Since this is a multiple-response question, *n* = 373, which is bigger than the number of cases in the data set (*n* = 100), in this case, the n value represents the total number of times a specific response was selected.

**Table 2:** Chi-square output on the effectiveness of COVID-19 response strategies and participants.

**Effectiveness of COVID-19 Response strategies**	**Yes**	**No**
Water supply	*n* = 2	*n* = 39
Refuse Disposal	*n* = 2	*n* = 63
Electricity supply	*n* = 1	*n* = 26
Sewerage collection and removal	*n* = 0	*n* = 29
Healthcare services	*n* = 2	*n* = 25
Community education programmes	*n* = 2	*n* = 16
Library with internet	*n* = 5	*n* = 14
Food resources	*n* = 7	*n* = 39
Information and resources	*n* = 0	*n* = 4
Adequate housing	*n* = 2	*n* = 15
Municipal roads	*n* = 2	*n* = 17
Freedom from crime	*n* = 4	*n* = 53
Community forums	*n* = 0	*n* = 2
Others	*n* = 0	*n* = 2

Source: Study Data SPSS Software.

Despite this significance, the majority of participants indicated a lack of effectiveness in the strategies employed by their municipality. Notably, one participant highlighted the lack of employment opportunities for residents of Walmer Township as a pressing unaddressed social need. The multiple-response format of the question allowed participants to select multiple options, resulting in a total response count (*n* = 373) exceeding the number of individual participants (*n* = 100). This suggests a broad range of responses but underscores the perception of insufficient municipal response to the pandemic.

### 3.4 Economic conditions exacerbated by COVID-19

There was no statistical significance difference between the participant's household employment status and the participant's response to COVID-19′s impact on the household's employment status (χ^2^ = 1.235; df = 4; *P* = 0.872). However, many participants reported yes when asked if COVID-19 affected the household's employment status. This result suggests that the observed differences could be due to chance rather than a meaningful relationship. However, it is noteworthy that many participants reported “yes” when asked if COVID-19 affected their household's employment status, highlighting that the pandemic's impact on employment was commonly experienced, even if the relationship to household employment status did not show statistical significance.

As concerns grew over employment precarity, 64% of the participants disclosed that COVID-19 had not affected their employment status, whilst 36% of the participants reported that COVID-19 had affected their employment status. Table 3 below represents an n value of the total number of participants that indicated that COVID-19 had impacted their household's employment status.

**Table 3:** Output on the COVID-19′s impact on participants' household employment status.

**COVID-19^′^s impact on employment status**	**Yes**	**No**
Lost my job	*n* = 4	*n* = 0
Working from home	*n* = 0	*n* = 0
Short time working	*n* = 20	*n* = 1
Working rotating shifts	*n* = 5	*n* = 0
Working part-time	*n* = 6	*n* = 0

Source: Study Data SPSS Software.

There was a statistically significant difference between participants' household income bracket and participants' response to COVID-19′s impact on household income (χ^2^ = 73.298; df = 5; *P* < 0.001). Moreover, many participants reported yes when asked if COVID-19 affected their household income. Whilst exploring COVID-19′s impact on participants' household income, participants were given the option of selecting multiple responses on how the pandemic has impacted their household and most participants reported their pension fund running out (Table 4). The effects of the pandemic on household income varied across different income groups. A substantial number of participants confirmed that COVID-19 affected their household income. When exploring the specific ways the pandemic impacted finances, participants highlighted multiple challenges, with the most frequently reported issue being the depletion of their pension funds. These findings underline the widespread and diverse financial disruptions caused by the pandemic, particularly among retirees and those relying on fixed income sources.

**Table 4:** Chi-square output on COVID-19′s impact on participants' household income bracket.

**COVID-19^′^s impact on household in-come**	**Yes**	**No**
Salary reduced	*n* = 48	*n* = 29
Working hours reduced	*n* = 48	*n* = 34
The pension fund ran out	*n* = 48	*n* = 51
Deceased breadwinner	*n* = 48	*n* = 45
No salary increment	*n* = 48	*n* = 36

Source: Study Data SPSS Software.

The Chi-Squared test did not reveal a statistically significant difference between participants' responses on food prices and how the NMBM can promote financial security (χ^2^ = 2.202; df = 5; *P* = 0.821). In this study, almost all (97%) participants indicated that they have experienced some economic hardships because of the rise in food prices. A minority (3%) of the participants indicated that the rise in food prices had not affected their households but, most participants (*n* = 77) reported no, when asked if the distribution of food parcels promotes financial security.

The findings suggest that while the rise in food prices is a widespread concern, the distribution of food parcels may not be perceived as an effective strategy to achieve long-term financial stability for these households. There was no statistical significance difference between participants' financial community development initiatives and participants' yes and no responses on financial community development (χ^2^ = 12.042; df = 12; *P* = 0.442). However, most participants reported that the most prevalent financial community development initiative is community stokvels (Table 5). This finding suggests that while statistical significance was not found in the responses, community stokvels play an important role in the financial practices in this community.

**Table 5:** Output on the prevalence of financial community development initiatives in the Walmer community.

**Financial community development initiatives**	**Yes**	**No**
Community stokvel	*n* = 47	*n* = 1
Teen pregnancy prevention	*n* = 11	*n* = 0
Computer literacy programmes	*n* = 9	*n* = 0
Substance abuse awareness	*n* = 20	*n* = 0
Fitness/wellness programmes	*n* = 22	*n* = 1
Community safety projects	*n* = 8	*n* = 0
Community gardening projects	*n* = 29	*n* = 0
Youth and education programmes	*n* = 18	*n* = 0
Maternity action	*n* = 0	*n* = 0
Free meal projects	*n* = 15	*n* = 2
Child daycare facilities	*n* = 1	*n* = 0
Sanitary drive	*n* = 1	*n* = 0
Winter blanket drive	*n* = 3	*n* = 0

Source: Study Data SPSS Software.

### 3.5 Perceptions and opinions on preparedness for the coronavirus

The themes that emerged from the opinions and perceptions of participants are “Altered family dynamics,” “Education,” “Employment,” and “Access to healthcare” (see Table 6). The COVID-19 brought about perilous adjustable conditions in families throughout the world. Studies that engaged issues of mental health consequences due to the quarantine period have reported increased altered family dynamics as the risk factor for anxiety, depression, post-traumatic stress disorder, and suicide (32). The COVID-19 pandemic highlighted another socio-economic concern, this digital divide affected students' access to quality education. Non-attendance and rotational attendance also meant that some scholars and students were denied access to feeding or nutritional programmes at school (33). In tertiary institutions, lack of access to data bundles could have disadvantaged economically excluded students.

**Table 6:** Nelson Mandela bay municipality preparedness for COVID-19.

**Themes**	**Sub-Themes**
Altered family dynamics	Bereavement
	Emotional detachment
	Improved personal hygiene
Education	Rotational learning
	School feeding programme
Employment	Retrenchment
	Unmet basic needs
	Domestic violence
Access to healthcare	Overcrowding of neighboring outpatient healthcare facilities
	Triage
	Defaulted treatment

Source: Study Data SPSS Software.

During the pandemic, an already bleak job market in South Africa loomed by a dark cloud of earnings loss, and breadwinners who were retrenched. “My father has lost his job and is no longer able to provide for us,” a participant commented on the economic consequence of the COVID-19 pandemic. This phenomenon not only worsens the living conditions of many households in this community but also unearths uncertainty and mental ill-health. With viruses, the most efficient response measure is in most cases antibacterial or antiviral medications.

In the international sector, COVID-19 has caused health system devastation and economic debasement (34). Systematic failures during the response to the rapidly contagious nature of the coronavirus came as a shock to an already pressed healthcare system of South Africa (34). The present pandemic if anything, served as a stark reminder that health is influenced by a wide range of social, economic, and political factors and involves much more than just physical wellbeing. COVID-19 has exposed the healthcare limitations of the “global north” and the “global south” alike (34, 35). These limitations range from a lack of hospital beds and medical equipment to glaring deficiencies in national surveillance systems, supply chains, and laboratory capacity. In summary, this contagion has burdened the already unequal and dilapidated health system in South Africa.

## 4 Discussion

Complementing the study findings, Cassese et al. (36) stipulate that women are less inclined to endorse COVID-19 conspiracy beliefs than men. This growing trend is attributed to the different health outcomes experienced by both men and women. Therefore, Cassese et al. (36) support the notion that gender has a major bearing on COVID-19 beliefs. A worrisome 14% of the respondents who reported that they do not believe that COVID-19 exists could have been based on the COVID-19 conspiracy theories circulated frequently on social media (37–39), studies have reported the negative impacts of social media and fake news on people's beliefs amidst the COVID-19 pandemic (40, 41). During the study's write-up, social gatherings were prohibited or kept at a minimum. Reinforcing this, October et al. (42) state that the COVID-19 novel has uniquely changed the dynamics of family and community relationships. Staying at home for some families strengthened familial relationships, but the opposite is true for some families, especially in families where tensions brew (42). This sort of variation in narratives speaks to the need to consider social disadvantages in South African communities (43).

Given that South Africa is no stranger to crime, it is unsurprising that the participants reported an increase in domestic violence during the COVID-19 lockdown. This assertion is supported by Baptiste-Roberts and Hossain (44), who explained that social disparities are often associated with elevated substance abuse and domestic violence levels. Suffering under the yoke of poverty, there is a high prevalence of domestic violence because of lifestyle pressures (45). Domestic violence is a heinous phenomenon that has become more pronounced since stringent lockdowns have been instituted (46). Concerning this study, 52% of the participants disclosed that levels of domestic violence worsened during the harsh lockdown. The surge in gender-based violence has been dubbed in the presidential addresses as a second pandemic that cannot be ignored (47).

A growing health concern in South Africa is mental health (48). The devastating impact of this contagion extends to mental health. The unsettling changes that ensued from the COVID-19 pandemic fueled fear, distress, and loneliness which are some of the mental health measures (35) Therefore, the results of many of the participants who were dependent on casual jobs as a source of income indicating that the source of income affected their mental health was not surprising. In the era of COVID-19, psychological stress emanated from the uncertainty surrounding the socio-economic aftermath of COVID-19 Seitz et al. (49) proclaimed that the burden of home-schooling, extended lockdown levels, rising death levels, elevated hardship, and unemployment precipitated further psychological stress. With the advent of COVID-19, it is evident that this pandemic has brought about a heightened awareness of illness and death (50). Mortality salience, in this sense, had a pernicious impact on participants' mental wellbeing. The findings are affirmed in a similar study conducted in the Soweto Township in South Africa whereby Kim et al. (32) reported that mental health illnesses such as depression and anxiety were found to increase during the pandemic. Moreover, Kim et al. (32) highlight that the high prevalence of mental illnesses was more pronounced amongst adults with childhood trauma and pre-existing adversities. While there were no statistically significant differences shown when participants were asked if COVID- 19 had impacted school performance, this does not mean the relationship does not exist. The results could imply that the relationship is too insignificant to worry about for these two variables as far as quantifying the data is concerned. Biggs and Tang (51) postulate that optimal learning is achieved when teaching and assessments complement students' learning outcomes. Again, statistically insignificant results do not necessarily mean socially insignificant (52). Given the distinctiveness of the South African education system, learning loss has been disproportionately experienced by students from vulnerable communities (53).

Indeed, most participants reported no when asked about social needs that they felt had been addressed by the municipality since COVID-19 started. This was not surprising, given that municipal efforts were directed at curbing the spread of the virus. Consequently, COVID-19 had overstretched limited municipal resources, which comprised the municipality's ability to meet this community's social needs.

Owing to restrictions on labor mobility COVID-19 has presented a damming phenomenon of labor scarcity, this study saw it fit to explore the financial woes precipitated by the pandemic. As expected, the study found that many participants reported yes when asked when asked if COVID-19 affected their household income. Consistent with our study findings, Schotte and Zizzamia (54) reported that residents of the Khayelitsha Township in Cape Town experienced economic volatility due to increased joblessness deepened the township's economic vulnerability. In fear of a new economic recession, Bambra et al. (35) poignantly explain that the economic consequences of COVID-19 have resulted in wage reductions and job losses.

Many such consequences include unpaid leave, mounting debt, and poverty levels. To add to this complexity, Schotte and Zizzamia (54) stipulated that among those with insufficient economic buffers were employees who could not apply for the Unemployment Insurance Fund (UIF) because they were ineligible due to short tenure.

Since COVID-19 has besieged global communities, economic sectors such as agricultural sectors have been disrupted. Sadly, this has inadvertently affected the sustainability of the food supply and the usual income of farm workers as, in most cases, they work on a no-work, no-pay basis. The findings that almost all (97%) participants indicated that they have experienced some economic hardships because of the rise in food prices are mirrored in a study by Hart et al. (55), which explored South Africa's experience of intensified hunger amid COVID-19. Drawing on the evidence from this study, Hart et al. (55) share that 64% of unemployed adults have shared a home with individuals who had gone to bed hungry during the lockdown period. Indeed, studies have reported community stokvels as the most viable way to curb financial worries in most African rural communities (56, 57). Therefore, the results of the majority of respondents reporting stokvels as the financial community development initiative was expected.

The section on perceptions and opinions on the coronavirus discussed the socio-economic impact of COVID-19 by homing in on emerging themes. Qualitative data allowed the exploration of untold consequences of COVID-19 within the context of the Walmer township. Preparedness in the backdrop of pre-existing socio-economic assailable contexts of a township hinders effective response. The fact that the COVID-19 virus is novel means new information, trends, and overall risk factors about the virus constantly evolving (58). The overall gap between disaster risk management and innovative socio-economic methodologies in the face of the COVID-19 pandemic is expressed in the nuances of people's narration of their lived experiences. Commonly, disaster responses have the potential of unfairly distributing risks (59). The stakeholder theory speaks to this phenomenon a great deal to some extent. Socioeconomic variables such as income level interplay with the impact of disasters. In a disaster affective context, the stakeholder theory is useful in determining a community's exposure to disaster risk. According to Zahran et al. (60), cited in Mojtahedi and Oo (61), reported that it is imperative to determine a community's susceptibility to experiencing injurious setbacks as the result of a specific disaster especially those caused by viruses. This therein contributes to the disaster intervention and management strategies employed to buffer the atrocities or negative outcomes caused by disasters in vulnerable communities.

The lack of significant relationships for socioeconomic conditions and municipal responses may reflect broader systemic factors, such as capacity issues, that municipal responses are unable to overcome. In cases where socioeconomic disparities are deeply rooted, local governments may struggle to respond due to financial issues and disparities that were exposed by the COVID-19 pandemic especially in developing countries across the world. This was evident in the 500 billion stimulus package for economic response that South Africa borrowed during the COVID-19 pandemic.

Municipal authorities play a critical role in protecting their communities during times of crisis, such as the COVID-19 pandemic. This study therefore based on the findings suggest specific strategies that can help improve socioeconomic protections for citizens during such events as follows: (1) Strengthen Social Safety Nets, based on the stokvels working in this community. (2) Support for Vulnerable Communities based on single participants reports on their effects of COVID-19. (3) Job Retention and Economic Recovery, this based on the results of pensioners funds running out. (4) Education and Digital Access, this recommendation is based on the A notable number of participants acknowledging that COVID-19 had impacted their school performance. By employing some of this strategies, municipal authorities can significantly improve socioeconomic protections for their citizens during the COVID-19 pandemic and contribute to the community's long-term resilience.

## 5 Study recommendations

Based on the findings this study suggests actionable recommendations for addressing the issues and improving COVID-19 preparedness in the Walmer community as follows: (1) Given the regression results showing deficits in COVID-19 preparedness in Walmer, there is a need for local authorities to reassess and strengthen their response strategies by increasing community education on COVID-19 prevention measures and improving communication channels for COVID-19 information. (2) Engaging gender in health campaigns as the higher belief in the existence of COVID-19 among males as compared to females suggests gender disparities in knowledge or attitudes. (3) Supporting families and relationships by developing support networks as the findings regarding single individuals experiencing disruptions in family dynamics and community relations indicate the need for emotional and social support programs. (4) Addressing domestic violence by launching community-based campaigns awareness on domestic violence during pandemics and provide accessible resources to victims as the reported increase in domestic violence across all age groups, calls for interventions to prevent and address this issue. (5) Addressing mental health challenges due to casual employment by providing mental support services, hotlines and counseling as many individuals who depend on casual jobs have reported mental health challenges due to loss of income during the pandemic. (6) Improving Education Support Systems by ensuring that students have access to online learning resources, as many participants indicate that COVID-19 has impacted their school performance, it is essential to address the education sector's challenges. (7) Improving the effectiveness of municipal COVID-19 response by gathering feedback from the community on the shortcomings of the response strategies that were employed during the pandemic. Given the dissatisfaction with the municipality's response, it's crucial to reassess and improve the strategies employed during the pandemic. (8) Supporting employment and household income by working with local businesses and the government to establish temporary financial relief programs as the disruption in employment and household income reported by many participants indicates a need for economic relief programs. (9) Addressing economic hardships from rising food prices through coordination with relevant stakeholders.

The almost universal experience of economic hardship due to rising food prices calls for immediate actions to alleviate food insecurity. (10) Strengthening community financial support networks by providing training and resources for community stokvels to better manage finances. (11) Addressing community perceptions of preparedness by creating community forums to deal with the issues raised by participants will address the themes of “Altered family dynamics,” “Education,” “Employment,” and “Access to healthcare” that emerged from participants' perceptions can help guide policy and community development. By taking these actionable steps, the Walmer community can improve its COVID-19 preparedness, mitigate the negative impacts of the pandemic, and build resilience against future health crises.

## 6 Conclusions

In conclusion, this study sheds light on the profound impact of the COVID-19 pandemic on the socio- economic landscape of the East Coast of South Africa. Despite commendable strides made toward achieving Sustainable Development Goals aimed at reducing vulnerabilities to socio-economic hardships, the pandemic has presented daunting challenges, disrupting progress and exacerbating existing inequalities. Through a meticulous mixed-method analysis employing descriptive statistics, chi-squared tests, and regression techniques, this research explored the intricate dynamics at play. While no significant relationship was found between the municipality's implementation of socio-economic protection measures and certain Likert scale questions, indicating a potential gap in policy execution, a notable discrepancy emerged concerning the effectiveness of COVID-19 response strategies and unmet community social needs. Although demographic variables did not exhibit statistically significant differences in relation to correlated variables, the findings underscore the urgency for targeted interventions to address the disparities exacerbated by the pandemic. It is imperative for policymakers, stakeholders, and communities to collaborate effectively in devising comprehensive strategies that mitigate the adverse effects of COVID-19 while advancing sustainable development objectives. The study fulfilled its purpose and has achieved its objectives by showing a case of a South African community where COVID-19 exacerbated the socio-economic disparities.

## Data Availability

The raw data supporting the conclusions of this article will be made available by the authors, without undue reservation.
